# Per- and Polyfluoroalkyl Substances (PFAS) in Sub-Antarctic Seabirds: Insights into Long-Range Transport and Bioaccumulation of Legacy and Replacement Chemicals

**DOI:** 10.1021/acsenvironau.5c00102

**Published:** 2025-10-03

**Authors:** Imogen R. Bailes, Richard A. Phillips, Jonathan L. Barber, Sara Losada, Lloyd S. Peck, Christopher Green, Andrew J. Sweetman

**Affiliations:** † Lancaster Environment Centre, 4396 Lancaster University, Library Avenue, Bailrigg, Lancaster LA1 4YQ, U.K.; ‡ 41820 British Antarctic Survey, Natural Environment Research Council, High Cross, Madingley Road, Cambridge CB3 0ET, U.K.; § 41843 Cefas (Centre for Environment, Fisheries and Aquaculture Science), Pakefield Road, Lowestoft NR33 0HT, U.K.; ∥ Defra (Department for Environment Food and Rural Affairs), Seacole Building, 2 Marsham Street, London SW1P 4DF, U.K.

**Keywords:** PFAS, pollution, biomonitoring, seabirds, Southern Ocean, Antarctic

## Abstract

Per- and polyfluoroalkyl substances (PFAS) are widespread environmental pollutants that can bioaccumulate in biota and cause a variety of adverse effects. Seabirds are useful bioindicators of pollutants in marine food webs because they are apex predators with broadly known diets and distributions, and concentrations in their tissues therefore reflect background exposure in particular regions and ecosystems. Concentrations of PFAS are high in seabirds in the Northern Hemisphere, but there have been few studies in the Southern Hemisphere, particularly in the sub-Antarctic, and these mostly involved a limited target list of PFAS. We detected 22 PFAS, of a target list of 39 compounds, in three species of procellariform seabirds (albatrosses and petrels) with different diets and migration strategies, sampled in two areas in the southwest Atlantic Ocean in 2004–2014. PFAS concentrations are reported for the first time in common diving petrels and white-chinned petrels. Concentrations in liver tissue varied significantly among species and years, with ΣPFAS ranging over 2 orders of magnitude from 0.08 to 7.5 ng/g (ww). Despite this variation in total concentrations, chemical contamination profiles were broadly similar, comprising mainly perfluorooctanesulfonic acid (PFOS) (∼80%) and perfluoroalkyl carboxylic acids (PFCAs) (∼15%), suggesting PFAS fingerprints are much the same despite the contrasting diets, trophic levels and distributions. This signature closely reflects mixtures found in south Atlantic waters and provides evidence of long-range transport of atmospheric precursors. Emerging compounds of concern including hexafluoropropylene oxide dimer acid (HFPO–DA), dodeceafluoro-3H-,4,8-dioxanonoate (ADONA), and short-chain perfluoroalkyl acids (PFAAs) were detected in some samples. This study provides evidence of contamination in biota and highlights the value of biomonitoring of remote environments.

## Graphical abstract

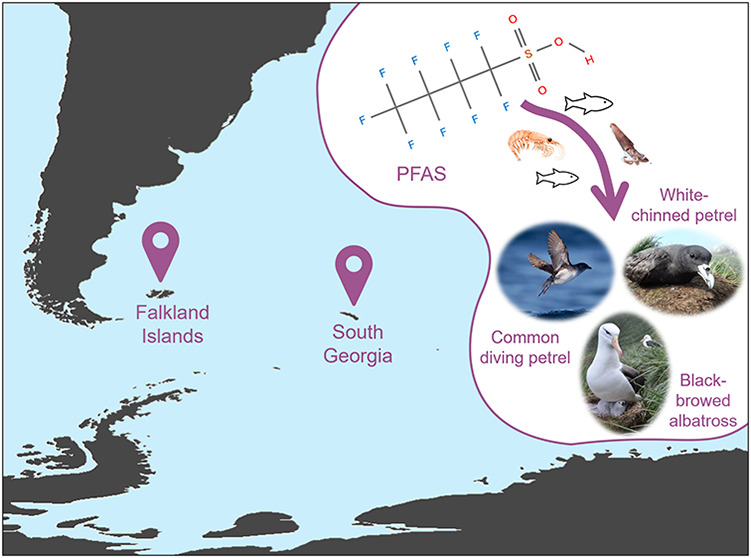

## Introduction

Per- and polyfluoroalkyl substances (PFAS) comprise a large group of chemicals that have the unusual properties of combined oil and water repellence, along with extreme stability, and hence are used in a wide range of industrial processes and consumer products. These chemicals can be problematic in the environment due to their persistence, toxicity, and ability to undergo long-range transport.^1–3^ Perfluorooctanesulfonic acid (PFOS), perfluorooctanoic acid (PFOA), perfluorohexanesulfonic acid (PFHxS), and long-chain perfluoroalkyl carboxylic acids (PFCAs) are restricted in the Annexes of the Stockholm Convention, which aims to protect the environment and human health from Persistent Organic Pollutants (POPs).^4^ POPs can be banned or restricted under the convention based on the criteria of persistence, bioaccumulation, toxicity and long-range transport. Further, the European Chemicals Agency is seeking to restrict the use and production of PFAS (defined as chemicals containing CF_2_) completely in Europe.^5^ As a result of these restrictions and other voluntary phase-outs, replacement chemicals are being detected with increasing frequency in the environment and biota.^6,7^ There is concern that these replacements could present as “regrettable substitutions” and do not pose a lower risk to organisms than the banned chemicals that they were designed to replace; such as was reported for hexafluoropropylene oxide dimer acid (HFPO–DA), having a higher toxic potency than PFOA in rats.^8^

PFAS can be released into the environment directly from industrial processes and from the use of consumer products. Release from wastewater treatment plants, the use of fire-fighting foams around airports and military bases, and the use of PFAS containing pesticides, are major sources of PFAS into the aquatic environment.^9^ Long-range transport of PFAS differs from that of traditional POPs as the former are more hydrophilic.^10,11^ Ionic PFAS are thought to be transported long distances in ocean currents,^12,13^ and neutral precursors are transported atmospherically before eventually degrading into terminal perfluoroalkyl acids (PFAAs).^11^ Antarctica was thought to be largely protected from long-range transport of PFAS because there is limited mixing of waters from the north to the south of the Antarctic Polar Front (APF).^14^ However, there is atmospheric input of PFAS to the continent,^15–17^ and so Antarctic environments are vulnerable to PFAS pollution. Point-source pollution from research stations has also been reported.^18^ Once in the Antarctic environment, PFAS can biomagnify through food chains, with dietary exposure and subsequent bioconcentration thought to be the most important pathways for accumulation,^19–22^ but maternal transfer has also been reported.^23^

Monitoring PFAS in wildlife is essential for understanding their global distribution as well as their bioaccumulation potential, and for detecting emerging chemicals of concern. It also supports regulatory decision making. Seabirds have been widely used in biomonitoring studies; they are ideal sentinel species due to their high trophic positions, long lifespans and known diets and distributions.^24^ Unlike lipophilic POPs, PFAS accumulate in protein-rich tissues such as liver and blood, which are therefore the matrices of choice for most studies.^24,25^ A myriad of adverse effects due to PFAS exposure have been reported in birds including disruptions to the endocrine, immune, and metabolic systems.^26–28^ The few previous Antarctic studies have reported concentrations up to 3.53 ng/g ww in liver tissue of seabirds,^29^ whereas higher concentrations have been reported in other tissue, up to 53 ng/g ww in plasma and up to 117 ng/g dw in eggs.^30,31^ However, studies investigating PFAS in seabird livers in the Antarctic region are limited, and studies focusing on other tissues often employ a restricted list of target compounds, including those already regulated globally.^4,29,31–33^ The aim of our study was to measure legacy and replacement PFAS concentrations in seabird species with varying diets and migration strategies to better understand exposure and subsequent biotic accumulation in this understudied region. Here, we analyzed a target list of 39 PFAS, including legacy and emerging compounds, in livers of three species of procellariform seabirds (albatrosses and petrels) sampled at the Falklands Islands and South Georgia, southwest Atlantic Ocean. The aims were to compare PFAS concentrations and profiles among years and species, and to determine whether the latter related to differences in distribution and prey types and trophic levels based on stable isotope ratios in the livers, and previous tracking and diet studies. Results are discussed in the context of environmental exposure, bioaccumulation, and the potential of different PFAS to undergo long-range transport.

## Materials and Methods

### Samples

Adult black-browed albatrosses (*Thalassarche melanophris*), white-chinned petrels (*Procellaria aequinoctialis*) and common diving petrels (*Pelecanoides urinatrix*) killed in fishing gear or in collisions with vessels were obtained between 2004 and 2014 from the Falkland Islands and South Georgia in October to February, and January to April, respectively. A total of 52 samples were collected (Table S1). These birds were stored frozen (−20 °C) initially, then dissected within a few months, and liver samples wrapped in aluminum foil and frozen before being transported back to the U.K. These samples were then stored at (−20 °C) for several years before analysis.

### PFAS Determination

Samples were processed at the Centre for Environment, Fisheries and Aquaculture Science (Cefas), Lowestoft, U.K., using a method described previously by O’Rourke et al.^34^ Additional targets compounds have since been added and so we describe the protocol again here. Samples were homogenized and then 1 g of sample was spiked with 20 μL of PFAS internal recovery working standard which contains a mixture of isotopically mass-labeled internal standards in methanol (0.2 ng/μL of each standard, see Supporting Information for full details) in a prerinsed polypropylene centrifuge tube. Samples were then extracted with 5 mL acetonitrile in an ultrasonic bath (15 min at room temperature), this process was repeated twice. Following each extraction, the supernatant solution was transferred to a new prerinsed centrifuge tube before being concentrated by a nitrogen blowdown to 1 mL. These extracts were then cleaned-up using 25 mg graphitized carbon (SupelClean ENVI-Carb 120/400, Supelco, Sigma-Aldrich, Stockholm, Sweden) and 50 μL of glacial acetic acid (99.7%, Sigma-Aldrich, Stockholm, Sweden). 500 μL of the cleaned-up extracts and 500 μL 4 mM aqueous ammonium acetate were then transferred to Eppendorf tubes and stored at 4 °C until the day of analysis. Prior to analysis, the extracts were transferred to injection vials, along with 10 μL of injection standard (see Supporting Information for details). The analysis of the suite of PFAS targets was done by isotopic dilution where available, and performed using an ultrahigh performance liquid chromatograph Vanquish Flex (Thermo Scientific, Massachusetts, United States) using an Acclaim RSLC C18 analytical column (2.2 μm particles, 100 mm × 2.1 mm, from Dionex, Thermo Scientific, Massachusetts, United States). A column Hypersil Gold (1.9 μm, 50 mm × 3 mm, from Thermo Scientific, Massachusetts, United States) was used as an isolator column. This UHPLC system was coupled to an Orbitrap Exploris TM 120 mass spectrometer (Thermo Scientific, Massachusetts, United States) using electrospray ionization (ESI) in the negative mode using full scan acquisition mode at a resolution of 120,000 and selected ion monitoring (SIM) at a resolution of 60,000. Mobile phases consisted of 2 mM ammonium acetate dissolved in methanol and 2 mM ammonium acetate dissolved in MeOH/water 2:98. The limit of detection (LOD) and limit of quantification (LOQ) for each compound were determined by three times and ten times the signal-to-noise ratio respectively during the validation study. To avoid extrapolation, the concentration equivalent to the calibration curve standard which has a signal-to-noise ratio of ten during the sample runs, is used as LOQ if greater than these. For quality assurance purposes, a blank and reference material were analyzed for every 10 samples. Method blanks were empty polypropylene centrifuge tubes, and the QC samples were NIST-1946 (Lake Superior fish tissue) and RM1 (in-house spiked mussel reference material). Control charts were used to evaluate the performance of the reference materials, aiming to repeat analysis of any batch if performance fell out of the control chart range. Blanks were below the detection or below the quantification limit for all batches and chemicals, so sample concentrations were not blank-corrected.

In addition, the method was validated at two levels (0.25 ng/g ww and 5 ng/g ww) using fish muscle as the matrix (Supporting Information). Recoveries obtained for the validation ranged from 63 to 119% for most chemicals and replicates. The exceptions were 8:8 PFPi and 10:2 FTSA which were not detected in the low-level spiked sample and showed lower and higher recoveries than expected in the high level sample (see Supporting Information).

The following compounds were targeted in this study: perfluorocarboxylic acids (PFCAs) C4–14, perfluorosulfonic acids (PFSAs) C4–10, perfluoroalkane sulfonamides C4, 6, and 8, 4:2, 6:2 and 8:2 fluorotelomer sulfonates and 3:3, 5:3, 7:3 fluorotelomer carboxylic acids, 6:6, 6:8 and 8:8 perfluoroalkyl phosphinates (PFPiAs) (full list in Supporting Information). Quantification of the targeted PFAS was based on isotopic dilution where labeled standards were available; when labeled standards were not available for a compound, a labeled standard with a similar retention time and/or functional group was used. The software TraceFinder 5.1 was used to quantify PFAS concentrations (Thermo Scientific, Massachusetts, United States). Calibration curves consisted of 11 calibration points with concentrations from 0.025 to 50 ng/mL. For quantification, curve type was adjusted to linear when possible, or to quadratic when instrumental response was not linear. Weighing was set to adjust to the lowest concentrations (1/x) and origin was ignored. Calibration curves obtained had *R* ^2^ > 0.995.

### Stable Isotope Analysis

Samples were lyophilized, ball milled and then 1 mg was weighed into tin capsules. The samples were combusted at 950 °C in an Elementar VARIO MICROcube Elemental Analyzer, and nitrogen and carbon stable isotopes (δ^15^N and δ^13^C) measured using an Isoprime100 Isotope Ratio Mass Spectrometer (IRMS). Standards (international or traceable) were IAEA 600 and USGS 41a for Nitrogen, IAEA CH6 and LEC-Acetanilide for Carbon and Elemental Microanalysis IRMS Protein standard.

δ^13^C values have been normalized mathematically to account for the lipid content according to eq 1.^35^

1 $$\delta^{13}C_{\mathrm{normalised}}=\delta^{13}C_{\mathrm{untreated}}-3.32+0.99\left(C:N\right)$$

### Data Treatment

Compounds below the limit of detection (LOD) were set to zero when calculating total PFAS values.^36^ Compounds detected above the LOD but below the limit of quantification (LOQ) were set to LOQ/2 for data visualization purposes as in comparable studies.^31,36^ Total PFAS values were found to be highly skewed as identified by visual inspections of histograms and the Shapiro-Wilk test; total PFAS data were therefore log transformed for statistical analysis. One-way ANOVAs were used to test for effects of species, sampling region (Falklands or South Georgia) and year on total PFAS concentrations (log-transformed) and stable isotope values (δ^15^N and δ^13^C). The models were found to satisfy assumptions of normality, homoscedasticity and leverage. Posthoc Tukey tests were conducted to generate pairwise comparisons for each species/sampling region/year group, for each model.

To evaluate associations between compounds and stable isotope values, a combination of spearman’s rank and kendall’s tau tests were conducted, as appropriate for noncensored and censored data (i.e., with nondetects). Spearman’s rank was chosen on the basis total PFAS concentrations violated assumptions of normality. Kendall’s tau correlations were generated for comparisons with censored chemicals using the cenken function in the package NADA in R.^37^ Only compounds detected in more than 50% of samples were selected for correlation analysis. A confidence interval of 95% was used to determine statistically significant correlations. All data analysis was conducted using R (version 4.3.2).

## Results and Discussion

This study is the first to report concentrations of a suite of 22 PFAS in seabirds in the Southern Ocean, including two species in which PFAS concentrations were analyzed for the first time. Studies suggest depuration half-lives in avian livers vary between 125 and 230 days for PFOS,^38,39^ and therefore it is probable that concentrations in bird livers sampled here represent accumulation over many months. However, in general there is a limited understanding of the toxicokinetics of other PFAS in seabirds, with studies in other species suggesting large differences in half-lives for various compounds.^40^ As such, we expect PFAS concentrations measured in our study to reflect accumulation over weeks to months. Given the timing of sampling (October to February at the Falklands, and January to April at South Georgia), this will reflect exposure to PFAS during and before the breeding season. Contamination profiles were strikingly similar between species, year and sampling region (Figure 1B), suggesting similar PFAS exposures from similar sources or similar accumulation, metabolism, and excretion patterns. This is despite large differences in foraging distributions and diet both among the three seabird species and between the two sampling regions. We explore possible explanations for these results in terms of the sources of PFAS in the Southern Hemisphere, the trophic ecology and distribution of the study species in the sampling regions, and any confounding factors.

**Figure 1:**
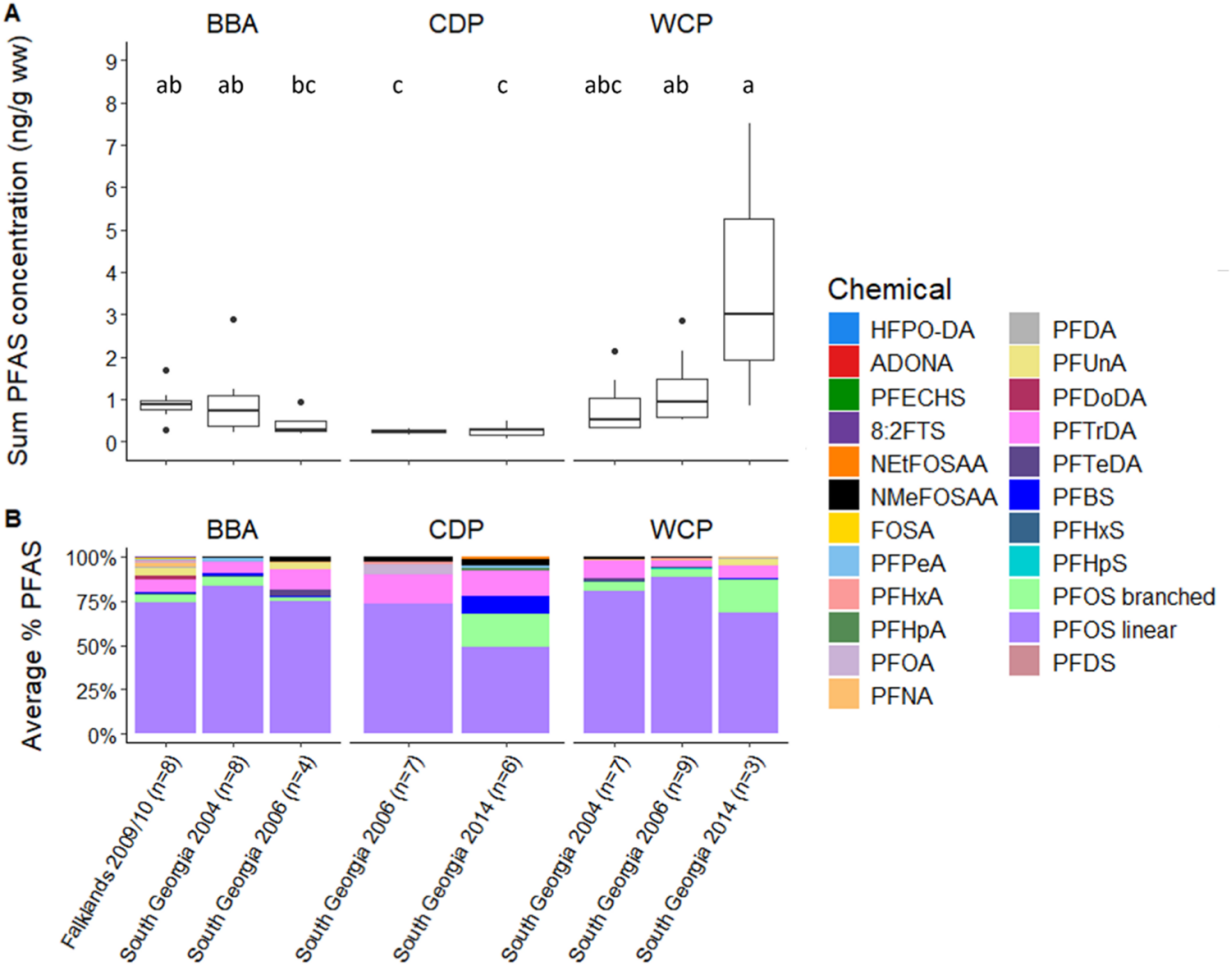
(A) Sum concentrations of 22 PFAS in individual liver tissues of black-browed albatrosses (BBA), common diving petrels (CDP) and white-chinned petrels (WCP) sampled in 2004 to 2014 at the Falklands Islands and South Georgia. (B) Average percentage contribution of various PFAS detected calculated on a ng/g basis, acronyms are detailed in Supporting Information. In the pairwise comparisons, groups that share the same superscript letters are not significantly different in mean PFAS concentrations according to a posthoc Tukey test following a one-way ANOVA on log-transformed data. *N* = 52.

We detected 22 of 39 target PFAS in the seabird liver samples, including legacy and replacement compounds (Figure 1A). Linear PFOS dominated profiles, averaging ∼74% of total PFAS detected. Branched PFOS were present in greater proportions in the samples collected in 2014, which were from common diving petrels and white-chinned petrels at South Georgia. Long-chain PFCAs were the next most dominant group of PFAS, particularly perfluorotridecanoic acid (PFTrDA), followed by perfluoroundecanoic acid (PFUnA) and perfluorotetradecanoic acid (PFTeDA). PFOA was detected in 15% of samples overall (Supporting Information). The next most dominant group were short-chain PFCAs. With the exception of PFOS, perfluorosulfonic acids (PFSAs) were present in small quantities in all sample groups. Perfluorobutanesulfonic acid (PFBS) was detected in several samples (19% overall) and contributed the largest proportion of PFSAs in common diving petrels at South Georgia in 2014 other than PFOS. Methyl-substituted perfluorooctane sulfonamidoacetic acid (NMeFOSAA) was detected in 29% of samples in small quantities, whereas ethyl-substituted perfluorooctane sulfonamidoacetic acid (NEtFOSAA) and perfluorooctanesulfonamide (FOSA) were rarely detected (∼2%). Replacement chemicals such as ADONA and GenX (HFPO–DA) were detected in one sample each: a black-browed albatross in 2009, and a white-chinned petrel in 2006, both at the Falklands. Concentrations were below the LOQ but above the LOD, and hence their presence was confirmed.

### PFAS and Seabird Ecology

Despite the similarities in profiles, absolute concentrations of total PFAS varied significantly among species: the lowest average concentrations were in common diving petrels (0.24 ± 0.03 ng/g ww), followed by black-browed albatrosses (0.82 ± 0.14 ng/g ww) and then white-chinned petrels (1.47 ± 0.39 ng/g ww) (Table S1). There were several outliers for total PFAS in black-browed albatrosses and white-chinned petrels, but not in common diving petrels in which the ranges were small in both sampling years (Figure 1A). Total concentrations varied among sampling regions and year (Figure 1A and Table S1). Total PFAS in one white-chinned petrel was particularly high (7.5 ng/g ww), in comparison to others in this study and was mainly dominated by linear and branched PFOS. Even when this value is excluded, white-chinned petrels still had the highest mean ΣPFAS (1.14 ± 0.2 ng/g ww). There was no significant temporal trend within species (Figure 1).

Not only were there no significant differences in PFAS concentrations between black-browed albatrosses sampled at South Georgia and the Falklands, but the contamination profiles for the birds from these two sampling regions were similar (Figure 1). Fewer PFCAs were detected in South Georgia individuals than in the Falklands (Figures 1 and 4). Black-browed albatrosses breeding in the Falklands are largely resident year-round on the Patagonian Shelf (Figure 2).^41^ Those breeding at South Georgia, however, feed at the Antarctic Polar Frontal Zone (APFZ) and the Brazil-Falklands Confluence during incubation, and at the APFZ to as far south as the South Orkney Islands when chick-rearing (Figure 2).^42,43^ This population then migrates during the nonbreeding season, mainly to the highly productive Benguela Upwelling off the coast of southern Africa, and a small minority to the Patagonian shelf or Australasia (Figure 2).^42–44^ There is, therefore, some spatial overlap, but analysis of mitochondrial DNA indicates that these two populations i.e., from South Georgia and the Falklands should be considered as separate evolutionary lineages.^45^ There are extensive fisheries on the Patagonian Shelf and Benguela Upwelling, and so black-browed albatrosses from both populations include fisheries discards as well as natural prey in their diet, which consists of crustaceans and cephalopods, particularly in the nonbreeding season.^46–49^ However, we would expect to see differences in both PFAS concentrations and contamination profiles given the contrasting distributions of these populations, particularly during the nonbreeding season. Environmental PFAS concentrations are much greater and include higher proportions of PFCAs in waters off the coast of South America compared to off the coast of southern Africa, where waters at the Benguela upwelling are dominated by PFOS.^50^

**Figure 2:**
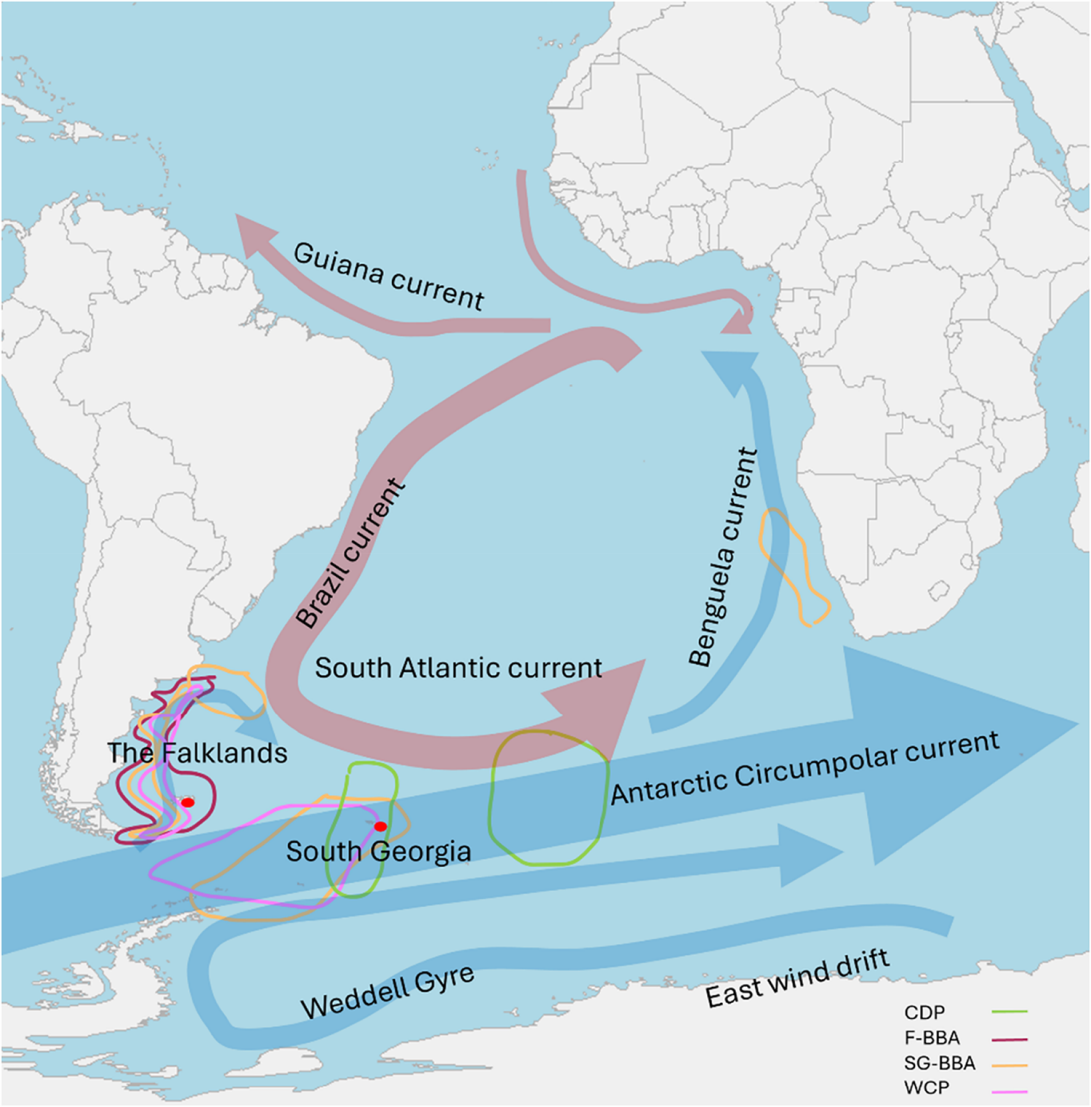
Representative map of major currents in the South Atlantic basin and Southern Ocean, with blue arrows representing the movement of cold water, and red warm water. Colored circles broadly represent the breeding and nonbreeding distributions of the three study species studied: common diving petrels (CDP), black-browed albatrosses from the Falklands (F-BBA) and South Georgia (SG-BBA) and white-chinned petrels (WCP).

White-chinned petrels from South Georgia feed on the Patagonian Shelf during the prelaying period, incubation and nonbreeding periods (Figure 2).^51^ During the chick-rearing period, they remain in Antarctic waters, foraging at the APFZ, local South Georgia shelf, central Scotia Sea or the South Orkney Islands.^51^ That they adopt this more oceanic distribution for only 3–4 months, and feed for the rest of the year on the Patagonian Shelf on a broadly similar diet to black-browed albatrosses^46,52^ might explain why their PFAS concentrations and profiles were similar to the black-browed albatrosses sampled in the Falklands. The much higher PFAS concentrations in white-chinned petrels sampled at South Georgia in 2014, and the higher proportion of branched PFOS in the profile (Figure 1) suggest that transport of PFAS to the Patagonian Shelf may have increased substantially in the following decade (see below).

The lowest mean PFAS concentrations in this study were mostly in common diving petrels sampled at South Georgia, although these were not significantly different from the other species in some regions in our earliest sampling years (Figure 1 and Table S1). Contamination profiles were broadly similar to the other species, apart from a greater proportion of branched PFOS in 2014 (Figure 1) and fewer detections of PFCAs (Figure 4). Common diving petrels are less wide-ranging than black-browed albatrosses and white-chinned petrels, feeding predominantly on the local shelf around South Georgia in the breeding season and for part of the nonbreeding period, and migrating 3000 km to oceanic waters to the east or northeast for the remainder of the year (Figure 4).^53,54^ Common diving petrels normally feed on copepods,^55^ hence its much lower trophic level than our other study species based on δ^15^N values (Phillips et al., 2009, this study). Both the lower trophic level and a foraging distribution distant from contamination sources on the South American continent are likely to explain the lower PFAS concentrations measured for this species.

In theory, we can use differences in stable isotope ratios to determine whether changes in diet, trophic level or distribution could explain changes in PFAS concentrations. However, links between stable isotope ratios and PFAS concentrations are inconsistent. Some studies found evidence for biomagnification of long-chain perfluoroalkyl carboxylic acids (PFCAs)^30,56^ and PFOS.^23,57^ However, others did not find correlations with δ^15^N values,^36,58^ or reported negative correlations with some PFCAs.^31,59,60^ In our study, stable isotope ratios varied among species, sampling regions and years (Figure 3 and Table S1), alluding to differences in trophic level and foraging distributions (Figure 3). However, it should be noted that liver stable isotope values reflect recent diet, i.e., weeks,^61^ whereas the PFAS concentrations reflect accumulation over months^38,39^ (see above).

**Figure 3:**
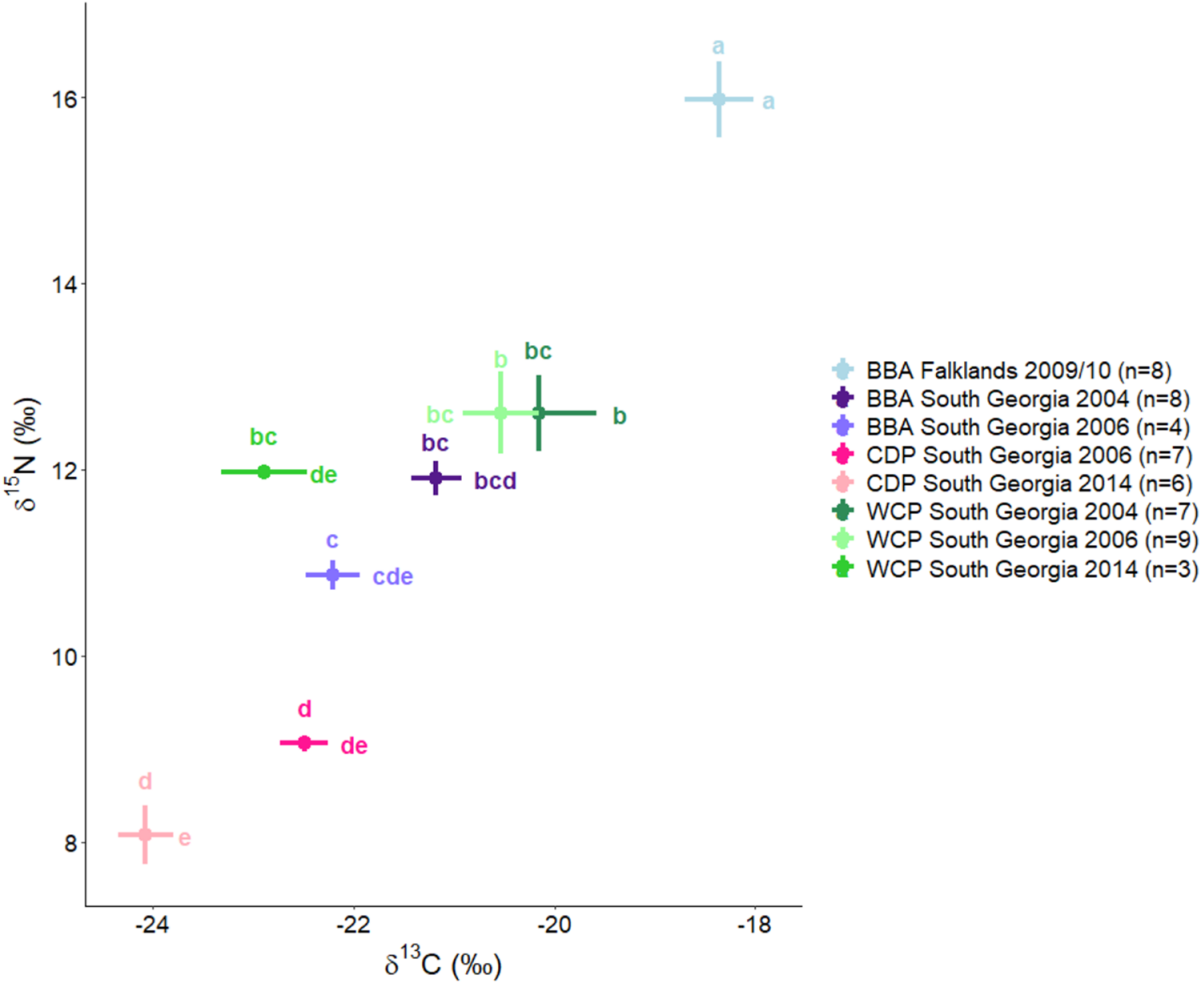
Mean ± SE δ^15^N and δ^13^C in liver tissue of black-browed albatrosses (BBA), common diving petrels (CDP) and white-chinned petrels (WCP) sampled in 2004 to 2014 at the Falklands Islands and South Georgia. δ^13^C values have been normalized mathematically to account for the lipid content.^35^ Letters indicate statistical groupings within each isotope measurement (δ^13^C horizontal, δ^15^N vertical); groups sharing letters are not significantly different (*p* > 0.05).

δ^15^N and δ^13^C values were consistently lower in common diving petrels than the other species. δ^15^N and δ^13^C values were broadly similar in white-chinned petrels and black-browed albatrosses from South Georgia, and significantly higher in black-browed albatrosses collected in 2009/10 from the Falklands (Table S1). Normalized δ^13^C values were more negative than expected in comparison to results for feathers in these species.^46,49,62–65^ However, this is the first study to measure stable isotopes in the livers of these species and therefore a direct comparison may not be appropriate, particularly as lower δ^13^C values have been reported in liver than feathers in other seabird species.^66^

There are no conventional diet studies of black-browed albatrosses during the nonbreeding season, but those in the breeding season indicate there can be extensive annual variation in the proportions of crustaceans, cephalopods and fish consumed.^48,49^ Replacement of crustaceans by fish and squid, which feed at higher trophic levels, in the diet of seabirds would explain higher δ^15^N values (*p* < 0.05) for birds sampled from the Falklands as compared to birds from South Georgia (Table S1 and Figure 3). However, in our study, the higher δ^15^N values did not translate to higher total PFAS concentrations, as might be expected for bioaccumulative compounds, but there were significant correlations between stable isotope ratios and some PFAS (Figure S1). Fish can effectively excrete PFAS through their gills as many of the compounds are hydrophilic, and therefore PFAS loadings are not as high in fish as in air-breathing organisms.^67^ Differences in δ^15^N values in black-browed albatrosses breeding at the Falklands and South Georgia may not indicate the birds are feeding at different trophic levels, but instead reflect differing baselines in each region (i.e., nitrogen isotope ratios at the base of the food web in the Patagonian shelf vs the APFZ).^68^ That could explain why PFAS concentrations did not differ. Lower δ^13^C values in common diving petrels than some other sampling groups (Figure 3) are indicative of foraging in Antarctic waters,^62,68^ where PFAS concentrations in prey could be lower, compared with south American food chains, because of reduced input from contaminated waters north of the Antarctic Polar Front (APF).^69^

### PFAS Sources in the Southern Hemisphere

PFOS is often the dominant compound among PFAS in seawater in the Southern Hemisphere, whereas PFOA is dominant in the Northern Hemisphere.^50,70,71^ This is likely due to continuing production of PFOA precursors such as fluorotelomer alcohols in the Northern Hemisphere vs continuing manufacture and use of PFOSF-based products that degrade to PFOS in the Southern Hemisphere.^70^ The active ingredient in the pesticide Sulfluramid is a PFAS, NEtFOSA, which is still used throughout South America for managing leaf-cutting ants that can have devastating impacts on soybean, maize, sugar and other crops.^72^ Brazil continues to produce this pesticide using PFOSF imported from China and exports it to other countries for the control of leaf-cutting ants.^73,74^ NEtFOSA can undergo both biotic and abiotic transformation in the environment to the intermediate FOSA and finally PFOS.^72,75,76^ The limited detection of FOSA in our samples (2%) could suggest rapid transformation to PFOS if the source is indeed Sulfluramid.^70^ Indeed, the detection of precursor compounds was limited in these samples, with those detected below the LOQ (Supporting Information) suggesting abiotic or biotic cycling before accumulation in liver tissue of the terminal compound. Exceptionally high concentrations of PFOS (3240 to 6560 pg/L) have been reported off the coast of Brazil.^50^ Although some authors suggest that the main contributor is Sulfluramid,^77^ others consider that its usage in Brazil is not sufficient to generate such high concentrations.^50^ Other contributions to total PFAS in the western South Atlantic may include wet and dry deposition of fluorinated precursors, as well as PFAAs used in the densely populated cities of Montevideo and Buenos Aires close to the Rio de la Plata estuary.^50,70^ There may also be input from elsewhere in major currents.^50^ PFAS concentrations as high as 1250 pg/L ΣPFAS^50^ have also been reported at the Benguela Upwelling which suggests currents are a key influence on spatial distribution, as production of PFAS is low in southern Africa.^78^ As such, the explanation for the far lower PFAS concentrations (∼20 pg/L) reported from pelagic regions of the South Atlantic^79^ is unclear, although perhaps the sampling was far from a major ocean current.

The strong easterly flow of the Antarctic Circumpolar Current is thought to limit the oceanic transport of PFAS from north to south of the APF.^14^ Atmospheric transport of precursors and subsequent wet deposition is therefore thought to be the main transport mechanism of PFAAs to the Antarctic continent.^11,15,16^ Amplification of PFAS in Antarctic coastal areas has also been reported as a result of penguin guano, sea-spray aerosol partitioning, and snowmelt.^15^ Varying concentrations of PFAS in Antarctic waters have been recorded, ranging from not detected,^3^ to ∼2000 pg/L around Deception Island in the South Shetland Islands.^15^ As such, the PFAS signal in white-chinned petrels and black-browed albatrosses that feed north of the APF during the breeding or nonbreeding seasons will predominantly reflect bioaccumulation of PFAS in food chains contaminated by PFAS from the Americas (Figure 4). We might expect that seabirds which feed around the Rio de la Plata estuary are likely to be impacted in particular by high concentrations of PFAS from Brazil.^70^ That their PFAS profiles and concentrations were similar to those of black-browed albatrosses from South Georgia which winter in the Benguela Upwelling seems likely to reflect oceanic transport from west to east in the South Atlantic Current (Figure 2), as the PFAS sources in Africa are much more limited.^78^ It is unclear why PFCAs are detected less frequently in seawater off the Benguela Upwelling and in seabirds that migrate there, but this may reflect various geochemical processes during long-range transport including temperature changes associated with upwelling, and/or a rainout effect from atmospherically transported fluorotelomers.^50^ Nonetheless, the overall similarity in PFAS profiles between black-browed albatrosses feeding around the Rio de la Plata and those wintering in the Benguela Upwelling suggests that long-range transport via the South Atlantic Current is a major driver of contamination patterns.

**Figure 4:**
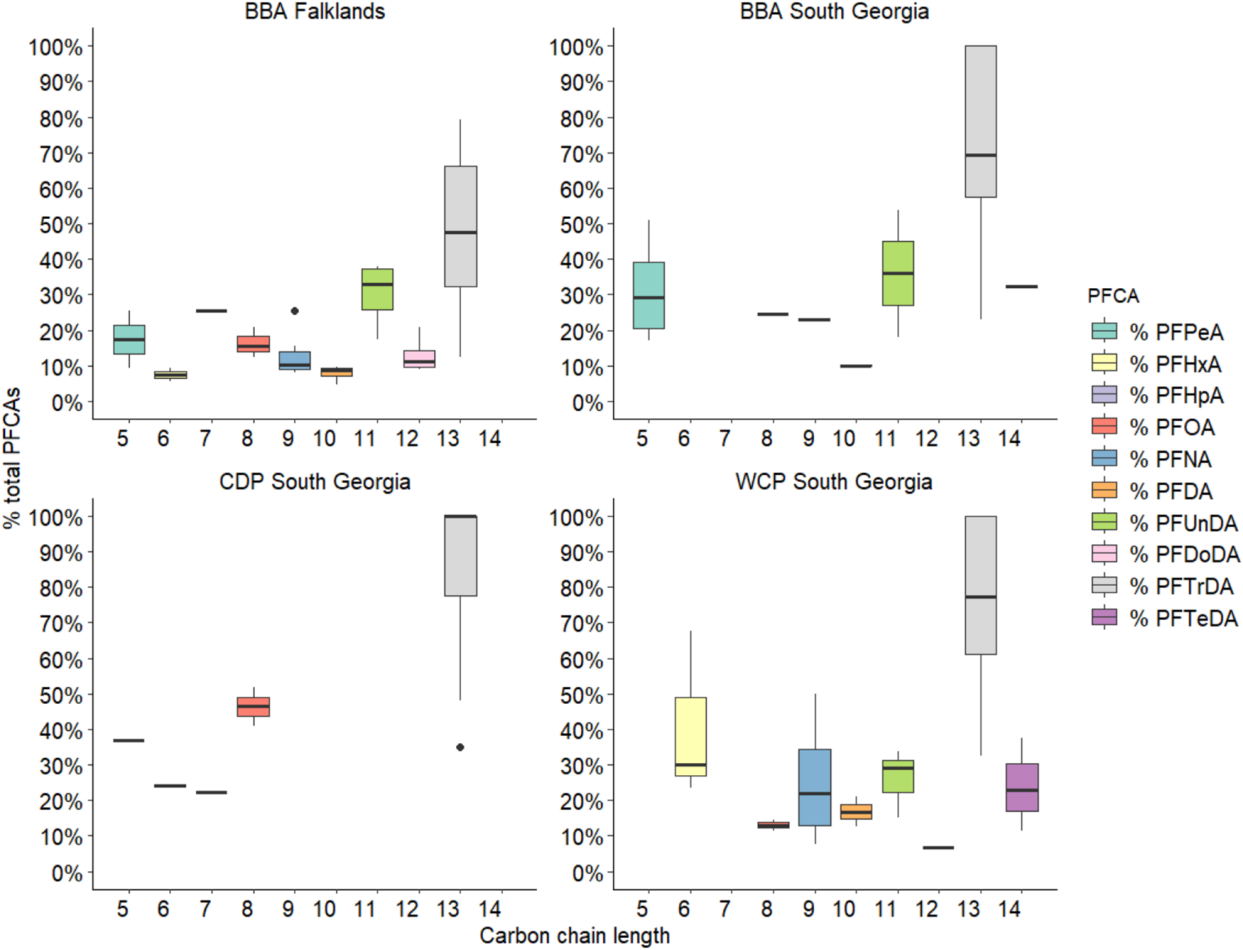
Percentage contribution of individual perfluoroalkyl carboxylic acids (PCFAs), ordered by carbon chain length, to the total PFCA concentration in liver tissues of black-browed albatrosses (BBA), common diving petrels (CDP) and white-chinned petrels (WCP) sampled in 2004 to 2014 at the Falklands Islands and South Georgia. Percentage contributions were calculated based on a ng/g basis. Only PFCAs detected above the limit of detection (LOD) are shown here, with boxplots representing the interquartile range, and range for individuals grouped by species and sampling location.

A greater proportion of branched PFOS were measured in samples in 2014 than in earlier years (Figure 1). This was associated with an increase in total PFAS concentrations in white-chinned petrels but not common diving petrels, suggesting concentrations around the Patagonian Shelf may have increased over time. This could indicate an increase in production volumes of the pesticide Sulfluramid manufactured by Brazil, which is produced using electrochemical fluorination and is characterized by linear and branched isomers. A higher quantity of branched NEtFOSA entering the environment could lead to increased quantities of branched PFOS in the environment due to environmental degradation and/or in vivo transformation and subsequent accumulation in seabirds. However, it would be useful to confirm this with samples from black-browed albatrosses in which we would also expect PFAS to have increased in more recent years. Branched PFAS are detected less frequently in wildlife compared to humans.^80,81^ Further, avian livers have previously been shown to enrich linear over branched PFOS isomers and so this is surprising result.^81^

Percentage contributions of the various PFCAs to total PFCA concentrations varied largely within species, and PFTrDA consistently dominated the profiles (Figure 4). Most targeted PFCAs were detected in black-browed albatrosses sampled from the Falklands, except for perfluorobutanoic acid (PFBA), and the majority of PFCAs were detected in white-chinned petrels and black-browed albatrosses from South Georgia. By comparison, few PFCAs were detected in common diving petrels, and these were mainly comprised of PFTrDA and shorter chain compounds such as PFOA, perfluorohexanoic acid (PFHxA), perfluoropentanoic acid (PFPeA) and perfluoroheptanoic acid (PFHpA) (Figure 4). Odd-chain homologues comprised higher contributions than even-chain homologues (Figure 4). This pattern was observed between PFCA chain lengths 6–7, 10–11 and 12–13 in black-browed albatrosses, and 8–9, 10–11, and 12–13 in white-chinned petrels (Figure 2).

The characteristic odd–even chain length pattern in PFCAs seen in the profiles of black-browed albatrosses and white-chinned petrels reflects an input from atmospheric precursors, which is well documented in mammals.^82,83^ The common diving petrels did not show this same pattern (Figure 4), even though some precursors have been detected in Antarctic air and their exposure to PFAS reflects atmospheric deposition and local cycling processes given they remain south of the APF year-round.^17^ This could reflect their lower PFAS concentrations in general, and therefore could be an artifact of detection limits. The lower trophic level of common diving petrels and potentially metabolic differences could also explain these findings (Figure 3).^31,84^ PFTrDA, a long-chain PFCA, was correlated with PFOS, suggesting a shared source or similar pathways of bioaccumulation in Antarctic food webs (Figure S1).

### Comparison with Other Studies

Roscales et al. reported the concentrations of ten PFCAs, five PFSAs and FOSA in the plasma of black-browed albatrosses from the Falkland Islands collected in 2009–2013 and found concentrations comparable to our results.^31^ While there were fewer targeted compounds, the exposure profiles were similar to this study in that linear PFOS comprised the majority of PFAS that were measured, but at a lower percentage (∼55%) than in our study (∼75%). On average, the remainder of the profiles were comprised mainly of long-chain PFCAs, some short-chain PFCAs and PFBS and FOSA. The lower percentage of PFOS measured by Roscales et al.^31^ could reflect a change in exposure over time, as their samples were collected up to 7 years later, and PFOS has decreased gradually in both environmental matrices and in seabirds in the Northern Hemisphere in recent years.^85^ Furthermore, plasma and liver may contain varying mixes of PFAS depending on the binding properties of the compounds and the availability of PFAS-binding molecules in each matrix. For example, very-low-density lipoproteins (VLDL) have an affinity for C_10_–C_15_ PFCAs, are synthesized in the liver and transported by the blood.^85,86^ The presence of these VLDLs could determine the concentration of long-chain PFCAs in different tissues.

Mean PFOS concentrations in livers of black-browed albatrosses bycaught in fisheries in the south Atlantic Ocean between 1992 and 1996 were far higher (3.53 ng/g; Tao et al.^29^) than in our samples (0.68 ± 0.11 ng/g ww). PFAS concentrations were also higher in gentoo penguins (*Pygoscelis papua*), brown skuas (*Stercorarius antarcticus*) and sooty shearwaters (*Ardenna griseus*) sampled at the Falkland Islands, and much higher in plasma of southern giant petrels plasma (*Macronectes giganteus*) at Gough Island.^31^ It is unclear why concentrations in this study were lower than in other studies, given trophic levels are comparable between species evaluated. Therefore, differences in concentrations are more likely to result from a temporal trend, differences in distribution or interspecific metabolism/excretion differences, rather than trophic level.^62,63,87^

Given the many studies that have documented a decrease in PFAS concentrations in the environment with increasing latitude,^3,50,70^ we might expect seabirds sampled at the Antarctic continent to be less contaminated than those in our study. However, that depends on year-round distribution, as many top predators are only present in the Southern Ocean during the austral summer and migrate to lower latitudes during the winter.^88^ That would explain why south polar skuas (*Stercorarius maccormicki*) sampled at several sites on the Antarctic continent have higher PFAS levels in various tissues,^1,33,89^ because this is a transequatorial migrant that accumulates most of the PFAS burden when feeding in the more contaminated Northern Hemisphere.^33,90,91^ Indeed, PFAS concentrations in seabirds breeding in the Northern Hemisphere are often an order of magnitude higher than in our study.^24^

### Emerging and Replacement Chemicals of Concern

Despite generally low concentrations of PFAS reported in this study, 22 PFAS were detected in the seabird livers, including emerging compounds of concern. Long-chain perfluoroalkyl carboxylic acids have recently been restricted under the Stockholm Convention,^4^ and were detected frequently in our samples. Perfluorotridecanoic acid (PFTrDA) was detected in all samples, while perfluorononanoic acid (PFNA) was detected in 23% of samples, perfluorodecanoic acid (PFDA) in 15%, perfluoroundecanoic acid (PFUnA) in 17%, perfluorododeacnoic acid (PFDoDA) in 10%, and perfluorotetradecanoic acid (PFTeDA) in 8% of samples (Supporting Information). Perfluoroalkyl ether carboxylic acids (PFECAs) HFPO–DA and ADONA were detected in one seabird liver each, in a black-browed albatross collected in 2009 and a white-chinned petrel collected in 2006, respectively. Concentrations were below the LOQ. PFECAs are used as replacements for banned chemicals such as PFOA because they were originally thought to be less bioaccumulative due to the ether bond.^92^ However, studies have found various PFECAs to have greater bioaccumulation potential and are possibly more toxic than PFOA,^8,93^ with several studies reporting their presence in biota (see ref 93 for a review and a recent report in gentoo penguins^94^). Perfluoroethylcyclohexanesulfonate (PFECHS) was detected below the LOQ in a black-browed albatross liver collected in 2004. PFECHS is a replacement candidate for PFOS and has previously been used in aircraft hydraulic fluids.^95^ 8:2 FTS (fluorotelomer sulfonate) was detected in a black-browed albatross liver collected in 2009 below the LOQ. Fluorotelomer sulfonates are PFOS replacements and are used in paints, coatings and industrial cleaning products.^96^ PFSA precursors were detected at various frequencies all below the LOQ: FOSA in one sample, NEtFOSAA in one sample and NMeFOSAA in 29% samples (see Supporting Information). Short-chain PFAAs, assumed to be less prone to bioaccumulation than the long-chain compounds^97^ have also been detected. These included PFPeA in 17% and PFBS in 19% of samples (Supporting Information).

The concentration of these chemicals is low, generally below the LOQ, but their presence was confirmed which is concerning given the remoteness of these sub-Antarctic samples. Furthermore, given PFECAs can preferentially accumulate in blood rather than liver tissue, the concentrations reported here likely underestimate the actual body burden.^98^ This finding warrants further investigation of emerging PFAS in contemporary Antarctic biota.

### Confounding Factors

The black-browed albatrosses and white-chinned petrels collected in this study were all killed in fisheries, as such there may be a bias toward those individuals that consume fish as a large proportion of their diet. This does not apply to the common diving petrels, which do not consume discards. Other factors such as protein and lipid levels in the sampled tissue could impact PFAS concentrations given their proteinophillic nature. Further, sex and exact age were unknown (although all samples were adults) and these factors may link to PFAS accumulation given their influence on at-sea distributions and hence exposure to pollutants.^42,99^ Links between sex and PFAS concentrations in wildlife are unclear, as some studies did not detect any effect,^100–102^ but others report differences,^103,104^ and maternal transfer.^23^ As discussed previously, there is limited research on the toxicokinetics of PFAS in avian livers^38,39^ and so the period of exposure reflected by liver concentrations is uncertain. Future studies determining the half-lives of each compound in seabird tissues would, therefore be of great value. Analysis of PFAS should also be conducted in more recent samples to assess whether global bans on some compounds have been effective and whether there is bioaccumulation of replacement chemicals.

### Perspective and Outlook

This study, as far as we are aware, is the first to report on an extensive target list of PFAS in sub-Antarctic seabirds, including two species in which PFAS concentrations were measured for the first time, and over spatial and temporal scales. We detected 22 PFAS with a method targeting 39 PFAS, including both legacy and emerging compounds across 52 samples from three seabird species, all of which contained PFAS. Emerging compounds were detected below the LOQ in a few samples. Given the toxicity of many PFAS is still unknown, this is of cause for concern, particularly given the many other known threats to these species, including from other contaminants such as mercury (Mills et al., 2022)^87^. The PFAS in their tissues likely originated from South America through a combination of cycling through the South Atlantic basin via oceanic currents, and from the degradation of atmospheric precursors, and then magnification in food chains.

Our study underlines the pressing need to monitor biota in remote environments to understand emerging risks as well as to assess bioaccumulation potential and long-range transport of both legacy and emerging pollutants. While regulatory measures have led to shifts in PFAS production and use in some regions, ongoing emissions continue to drive global contamination, including in the southern Atlantic Ocean and remote environments. Strengthening global policy and regulation, alongside consistent monitoring and further research will be critical in protecting these remote ecosystems from pollution, particularly given the other ongoing threats from climate change and biodiversity loss.

## Supplementary Material





## Notes

Additional details on methodology including full target suite and validation experiment, mean values results table and statistical groupings, correlation results (PDF)
PFAS concentrations for each sample, frequency of detection, recoveries for this study and validation experiment (XLSX)
