# Supplementary material for: Per- and Polyfluoroalkyl Substances (PFAS) in Sub-Antarctic Seabirds: Insights into Long-Range Transport and Bioaccumulation of Legacy and Replacement Chemicals
Source: ACS Environ Au. 2025 Oct 3;5(6):603–15. doi: 10.1021/acsenvironau.5c00102 (PMC12635936; doi:10.1021/acsenvironau.5c00102)
Supplement: Supplementary file 1 [file vg5c00102_si_001.pdf]

Per- and polyfluoroalkyl substances (PFAS) in sub-Antarctic seabirds: insights into long-range transport and bioaccumulation of legacy and replacement chemicals

Imogen R. Bailes[a], Richard A. Phillips[b], Jonathan L. Barber[c], Sara Losada[c], Lloyd Peck[b], Christopher Green[d], and Andrew J. Sweetman[a].*

[a] Lancaster Environment Centre, Lancaster University, Library Avenue, Bailrigg, Lancaster, LA1 4YQ, UK.

[b] British Antarctic Survey, Natural Environment Research Council, High Cross, Madingley Road, Cambridge, CB3 0ET, UK.

[c] Cefas (Centre for Environment, Fisheries and Aquaculture Science), Pakefield Road, Lowestoft, NR33 0HT, UK.

[d] Defra (Department for Environment Food and Rural Affairs), Seacole Building, 2 Marsham Street, London, SW1P 4DF, UK.

*Email: a.sweetman@lancaster.ac.uk

Methods

Internal recovery working standard

- **MPFAC-24ES Labelled mixture solution** of $^{13}C_{4}$-perfluorobutanoic acid (MPFA or $^{13}C_4$PFBuA), $^{13}C_{5}$-perfluoropentanoic acid (M5PFPeA or $^{13}C_5$PFPeA), $^{13}C_{5}$-perfluorohexanoic acid (M5PFHxA or $^{13}C_5$PFHxA), $^{13}C_{4}$-perfluoroheptanoic acid (M4PFHpA or $^{13}C_4$PFHpA), $^{13}C_{8}$-perfluorooctanoic acid (M8PFOA or $^{13}C_8$PFOA), $^{13}C_{9}$-perfluorononanoic acid (M9PFNA or $^{13}C_9$PFNA), $^{13}C_{6}$-perfluorodecanoic acid (M6PFDA or $^{13}C_6$PFDcA), $^{13}C_7$PFUnA -perfluoroundecanoic acid (M7PFUdA

or $^{13}C_7$PFUnA), $^{13}C_2$PFDoDA -perfluorododecanoic acid (MPFDoA or $^{13}C_2$PFDoDA), $^{13}C_{2}$-perfluorotetradecanoic acid (M2PFTeDA or $^{13}C_2$PFTeDA), $^{13}C_{8}$-perfluorooctane sulfonamide (M8FOSA or $^{13}C_8$PFOSA), d_{3}-n-methyl perfluorooctanesulfonamidoacetic acid (D3-N-MeFOSAA or d_3NMeFOSAA), d_{5}-n-ethyl perfluorooctanesulfonamidoacetic acid (D5-NetFOSAA or d_5NEtFOSAA), $^{13}C_{3}$-perfluorobutane sulfonic acid (M3PFBS or $^{13}C_3$PFBuS), $^{13}C_{3}$-perfluorohexane sulfonic acid (M3PFHxS or $^{13}C_3$PFHxS), $^{13}C_{8}$-perfluorooctane sulfonic acid (M8PFOS or $^{13}C_8$PFOS), $^{13}C_{2}$-1H,1H,2H,2H-perfluorohexanesulfonic (M2 4:2FTS or $^{13}C_2$4:2FTS), $^{13}C_{2}$-1H,1H,2H,2H-perfluorooctanesulfonic acid (M2 6:2FTS or $^{13}C_2$6:2FTS), $^{13}C_{2}$-1H,1H,2H,2H-perfluorodecanesulfonic acid (M2 8:2FTS or $^{13}C_2$8:2FTS) (at ~ 1 µg/mL); 2 x 1.2 mL (Wellington Standards, Guelph, Canada).

- 2,3,3,3,-Tetrafluoro-2-(1,1,2,2,3,3,3,-heptafluoropropoxy)-13C3-propanoic acid (M3HFPO-DA) and 2-perflorodecyl (1,2 13C2) ethanoic acid (MFDEA).

PFAS injection standard working

- **MPFAC-C-IS Injection mixture solution** of $^{13}C_3$-perfluorobutanoic acid (M3PFBA or $^{13}C_3$PFBuA), $^{13}C_{2}$-perfluorooctanoic acid (M2PFOA or $^{13}C_2$PFOA), $^{13}C_{2}$-perfluorododecanoic acid (M2PFDA or $^{13}C_2$PFDcA) and $^{13}C_4$-perfluorooctane sulfonic acid (M4PFOS or $^{13}C_4$PFOS) (2 µg/mL); 1.2 mL (Wellington Standards, Guelph, Canada).

Table 1. Full suite of chemicals targeted in PFAS method.

Chemical name	*Abbreviation*	*Carbon number*	*LOQ (ng/g)*	*Analytical standard*
Perfluoroalkyl carboxylic acids (PFCA)				
Perfluorobutanoic acid	*PFBA*	*4*	*0.05*	*13C4-PFBA*
Perfluoropentanoic acid	*PFPeA*	*5*	*0.05*	*13C5-PFPeA*
Perfluorohexanoic acid	*PFHxA*	*6*	*0.05*	*13C5-PFHxA*
Perfluoroheptanoic acid	*PFHpA*	*7*	*0.05*	*13C4-PFHpA*
Perfluorooctanoic acid	*PFOA*	*8*	*0.024*	*13C8-PFOA*
Perfluorononanoic acid	*PFNA*	*9*	*0.024*	*13C9-PFNA*
Perfluorodecanoic acid	*PFDA*	*10*	*0.024*	*13C6-PFDA*
Perfluoroundecanoic acid	*PFUnA*	*11*	*0.024*	*13C7-PFUnDA*
Perfluorododecanoic acid	*PFDoDA*	*12*	*0.024*	*13C2-PFDoDA*
Perfluorotridecanoic acid	*PFTrDA*	*13*	*0.024*	*13C2-PFDoDA*
Perfluorotetradecanoic acid	*PFTeDA*	*14*	*0.024*	*13C2-PFTeDA*
Perfluoroalkyl sulfonic acids (PFSA)				
Perfluorobutane sulfonic acid	*PFBS*	*4*	*0.022*	*13C3-PFBS*
Perfluoropentane sulfonic acid	*PFPeS*	*5*	*0.048*	*13C3-PFBS*
Perfluorohexane sulfonic acid	*Linear PFHxS*	*6*	*0.02*	*13C3-PFHxS*

Perfluorohexane sulfonic acid	*Branched PFHxS*	*6*	*0.008*	*13C3-PFHxS*
Perfluoroheptane sulfonic acid	*PFHpS*	*7*	*0.024*	*13C3-PFHxS*
Perfluorooctane sulfonic acid	*Linear PFOS*	*8*	*0.018*	*13C8-PFOS*
Perfluorooctane sulfonic acid	*Branched PFOS*	*8*	*0.01*	*13C8-PFOS*
Perfluorononane sulfonic acid	*PFNS*	*9*	*0.024*	*13C8-PFOS*
Perfluorodecane sulfonic acid	*PFDS*	*10*	*0.024*	*13C8-PFOS*
Perfluoroalkane sulfonamides (FASA)				
Perfluorobutane sulfonamide	*FBSA*	*4*	*0.024*	*13C8-PFOSA*
Perfluorohexane sulfonamide	*FHxSA*	*6*	*0.025*	*13C8-PFOSA*
Perfluorooctane sulfonamide	*FOSA*	*8*	*0.024*	*13C8-PFOSA*
Fluorotelomer sulfonates (FTS)				
1H,1H,2H,2H-Perfluorohexanesulfonic acid	*4:2FTS*	*6*	*0.046*	*13C2-4:2FTS*
1H,1H,2H,2H-Perfluorooctanesulfonic acid	*6:2FTS*	*8*	*0.048*	*13C2-6:2FTS*
1H,1H,2H,2H-Perfluorodecanesulfonic acid	*8:2FTS*	*10*	*0.048*	*13C2-8:2FTS*
Fluorotelomer carboxylic acids (FTCAs)				
3-Perfluoropropyl propanoic acid	*FPrPA 3:3*	*6*	*0.05*	*13C4-PFBA*
3-Perfluoropentyl propanoic acid	*FPePA 5:3*	*8*	*0.05*	*13C8-PFOA*
3-Perfluoroheptyl propanoic acid	*FHpPA 7:3*	*10*	*0.05*	*13C4-PFHpA*
Perfluoroalkyl phosphinic acids (PFPis)				
6:6 Perfluorophosphinic acid	*6:6 PFPi*	*12*	*0.05*	*13C7-PFUnDA*
6:8 Perfluorophosphinic acid	*6:8 PFPi*	*14*	*0.05*	*13C2-PFDoDA*
8:8 Perfluorophosphinic acid	*8:8 PFPi*	*16*	*0.05*	*13C2-PFTeDA*
Other PFAS compounds				
N-ethyl perfluorooctanesulfonamidoacetic acid	*NEtFOSAA*	*8*	*0.05*	*d5-NEtFOSAA*
N-methyl perfluorooctanesulfonamidoacetic acid	*NMeFOSAA*	*8*	*0.05*	*d3-NMeFOSAA*
Hexafluoropropylene oxide-dimer acid	*HPFO-DA*	*6*	*0.05*	*13C3-HPFOA-DA*
Sodium dodecafluoro-3H-4,8-dioxanonanoate	*ADONA*	*7*	*0.024*	*13C8-PFOS*
Perfluoroethylcyclohexane sulfonate	*PFECHS*	*8*	*0.048*	*13C8-PFOS*
Potassium-9-chlorohexadecafluoro-3-oxanonane-1-sulfonate	*9Cl-PF3ONS/6:2 Cl-PFESA (F53 Major)*	*8*	*0.046*	*13C8-PFOS*
Potassium-11-chloroeicosafluoro-3-oxaundecane-1-sulfonate	*11Cl-PF3OUdS/8:2 Cl-PFESA (F53 Minor)*	*10*	*0.048*	*13C8-PFOS*

Validation experiment

The full method was validated at two levels (0.25 ng/g ww and 5 ng/g ww) using fish muscle as the matrix. The recoveries obtained during validation ranged from 63% to 119% for most chemicals and replicates. The exceptions were 8:8PFPi and 10:2 FTSA, which were not detected in the low level spiked sample and showed lower and higher recoveries than expected in the high level sample (see Supporting Information.xlsx). As regular part of the quality assurance of the method, the laboratory takes part twice a year in the interlaboratory study Quasimeme (Wepal-Quasimeme, Wageningen Research, the Netherlands). The samples analysed include biota and sediment samples, obtaining good results for the validated method every round.

Results

Table S1. Mean ± standard error, and pairwise comparisons evaluated by a one-way ANOVA of Sum PFAS, $\delta^{15}N$ and $\delta^{13}C$ in livers of black-browed albatrosses (BBA), common diving petrels (CDP) and white-chinned petrels (WCP) sampled in 2004 to 2014 at the Falklands Islands and South Georgia. In the pairwise comparisons, groups that share the same superscript letters are not significantly different according to a post-hoc Tukey test following a one-way ANOVA. Sum PFAS data were log-transformed before statistical analysis. Individual PFAS concentrations are detailed in the Supporting Information. N=52.

				MEAN ± STANDARD ERROR			PAIRWISE COMPARISONS		
SPECIES	Sampling region	Year	n	$\delta^{15}N$ (‰)	$\delta^{13}C$ (‰)	Sum PFAS (ng/g ww)	$\delta^{15}N$ (‰)	$\delta^{13}C$ (‰)	Sum PFAS (ng/g ww)
BLACK-BROWED ALBATROSS	Falklands	2009/ 2010	8	15.98 ± 0.41	-18.36 ± 0.34	0.89 ± 0.14	a	a	ab
	South Georgia	2004	8	11.91 ± 0.19	-21.17 ± 0.25	0.93 ± 0.3	bc	bcd	ab
	South Georgia	2006	4	10.87 ± 0.16	-22.2 ± 0.26	0.43 ± 0.17	c	cde	bc
**BLACK-BROWED ALBATROSS**	**All**	**All**	**20**	**13.33 ± 0.53**	**-20.25 ± 0.4**	**0.82 ± 0.14**			
	South Georgia	2006	7	9.06 ± 0.09	-22.49 ± 0.24	0.23 ± 0.02	d	de	c

COMMON DIVING PETREL	South Georgia	2014	6	8.08 ± 0.32	-24.07 ± 0.28	0.25 ± 0.06	d	e	c
**COMMON DIVING PETREL**	**All**	**All**	**13**	**8.6 ± 0.2**	**-23.22 ± 0.29**	**0.24 ±0.03**			
WHITE-CHINNED PETREL	South Georgia	2004	7	12.6 ± 0.41	-20.15 ± 0.57	0.82 ± 0.27	bc	b	abc
	South Georgia	2006	9	12.61 ± 0.44	-20.53 ± 0.38	1.22 ± 0.27	b	bc	ab
	South Georgia	2014	3	11.97 ± 0.05	-22.89 ± 0.43	3.78 ± 1.96	bc	de	a
**WHITE-CHINNED PETREL**	**All**	**All**	**19**	**12.5 ± 0.26**	**-20.8 ±0.35**	**1.47 ± 0.39**			

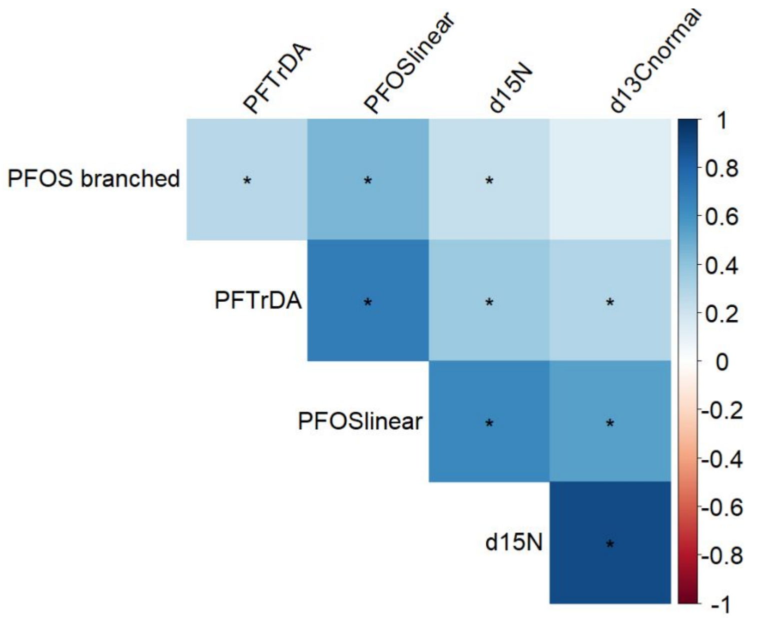

Figure S1. Correlation values for PFAS compounds detected in more than 50% of samples, and $\delta^{15}N$ and $\delta^{13}C$ in liver tissue of black-browed albatrosses (BBA), common diving petrels (CDP) and white-chinned petrels (WCP) sampled in 2004 to 2014 at the Falklands Islands and South Georgia. Asterisks indicate statistically significant (95% confidence interval) correlations.

There were positive significant correlations between concentrations of all PFAS compounds that were detected in 50% of more of samples, branched and linear PFOS and PFTrDA (Figure S1). $\delta^{15}N$ and $\delta^{13}C$ values were

also significantly correlated with PFAS compounds, except for the correlation between $\delta^{13}C$ values and branched PFOS (Figure S1).
